# Detoxification-related gene expression accompanies anhydrobiosis in the foliar nematode (*Aphelenchoides fragariae*)

**DOI:** 10.21307/jofnem-2020-047

**Published:** 2020-05-25

**Authors:** Zhen Fu, Paula Agudelo, Christina E. Wells

**Affiliations:** 1 School of Agricultural, Forest, and Environmental Sciences, Clemson University, Clemson, SC, 29634; 2 Department of Entomology, Washington State University, Pullman, WA, 99164; 3 Department of Biological Sciences, Clemson University, Clemson, SC, 29634

**Keywords:** Anhydrobiosis, Detoxification, Extended life span, Foliar nematode, RNA-seq, Transcriptome

## Abstract

The foliar nematode (*Aphelenchoides fragariae*) is a quarantined pest that infects a broad range of herbaceous and woody plants. Previous work has demonstrated its remarkable ability to survive rapid and extreme desiccation, although the specific molecular mechanisms underlying its anhydrobiotic response have not been characterized. The authors used RNA sequencing and *de novo* transcriptome assembly to compare patterns of gene expression between hydrated and 24-hr desiccated nematodes. In total, 2,083 and 953 genes were significantly up- and downregulated, respectively, in desiccated nematodes. Of the 100 annotated genes with the largest positive fold-changes, more than one third encoded putative detoxification-related proteins. Genes encoding enzymes of Phase I and Phase II detoxification systems were among the most strongly upregulated in the transcriptome, including 35 cytochrome p450s, 23 short chain dehydrogenase/reductases, 5 glutathione-S-transferases, and 22 UDP-glucuronosyltransferases. Genes encoding heat shock proteins, unfolded protein response enzymes, and intrinsically disordered proteins were also upregulated. Anhydrobiosis in *A. fragariae* appears to involve both strategies to minimize protein misfolding and aggregation, and wholesale induction of the cellular detoxification machinery. These processes may be controlled in part through the activity of forkhead transcription factors similar to *Caenorhabditis elegans*’ *daf-16*, a number of which were differentially expressed under desiccation.

The foliar nematode, *Aphelenchoides fragariae* (Ritzema Bos) Christie, is a quarantined endo- and ecto-parasite that infects a broad range of herbaceous and woody host plants (Jagdale and Grewal, 2002; McCuiston et al., 2007; Kohl et al., 2010; Fu et al., 2012; Sanchez-Monge et al., 2015). It enters plant leaves through wounds and stomata, where it feeds on mesophyll cells and causes characteristic vein-delimited lesions that reduce the appearance and marketability of ornamental plants (Wallace, 1959; Kohl et al., 2010; Fu et al., 2012). Previous work in our lab has demonstrated its ability to survive extreme desiccation, although the molecular mechanisms underlying its desiccation tolerance have not been characterized (Fu et al., 2012).

The foliar nematode survives overwinter in soil, dormant buds, and abscised leaves, where its desiccation tolerance allows it to endure freezing temperatures and low relative humidity (Jagdale and Grewal, 2006). Like a number of other nematode species, *A. fragariae* is capable of entering an anhydrobiotic state in which it loses >99% of detectable body water and suspends both metabolism and aging (Crowe and Madin, 1975; Crowe et al., 1992). Anhydrobiosis has been demonstrated in several Antarctic nematode species (Wharton, 2003), as well as animal parasitic, plant parasitic, and entomopathogenic nematodes (Patel et al., 1997; Wharton and Worland, 2001; Karim et al., 2009; Anbesse et al., 2013; Nimkingrat et al., 2013; Chylinski et al., 2014; Shapiro-Ilan et al., 2014). An extreme example is the stem and bulb nematode *Ditylenchus dipsaci*, which has been shown to survive up to 23 yr in dry storage (Fielding, 1951).

We have previously documented the remarkable anhydrobiotic behavior of *A. fragariae*, which displays significantly greater survivorship and faster recovery from desiccation than the model anhydrobiotic nematode, *Aphelenchus avenae* (Fu et al., 2012). In response to dehydration, *A. fragariae* aggregated into compact clusters and increased the expression of glutaredoxin and trehalose phosphate synthase genes (Fu et al., 2012). However, it is not entirely clear what other molecular mechanisms are involved in foliar nematode desiccation. Here, we report the *de novo* assembly of an *A. fragariae* transcriptome constructed from well-hydrated and 24-hr-desiccated nematodes. The most striking result was the wholesale upregulation of multiple genes encoding Phase I and II detoxification enzymes: numerous cytochrome p450s (CYPs), short chain dehydrogenase/reductases (SDRs), UDP-glucuronosyltransferases (UGTs), and glutathione-S-transferases (GSTs), as well as related multi-drug resistance transporters. Heat shock proteins, enzymes of the unfolded protein response, and intrinsically disordered proteins (IDPs) were also strongly induced by dehydration, suggesting that the prevention and mitigation of protein damage is a central feature of *A. fragariae*’s desiccation response.

## Materials and methods

### Nematodes

**Figure 1:**
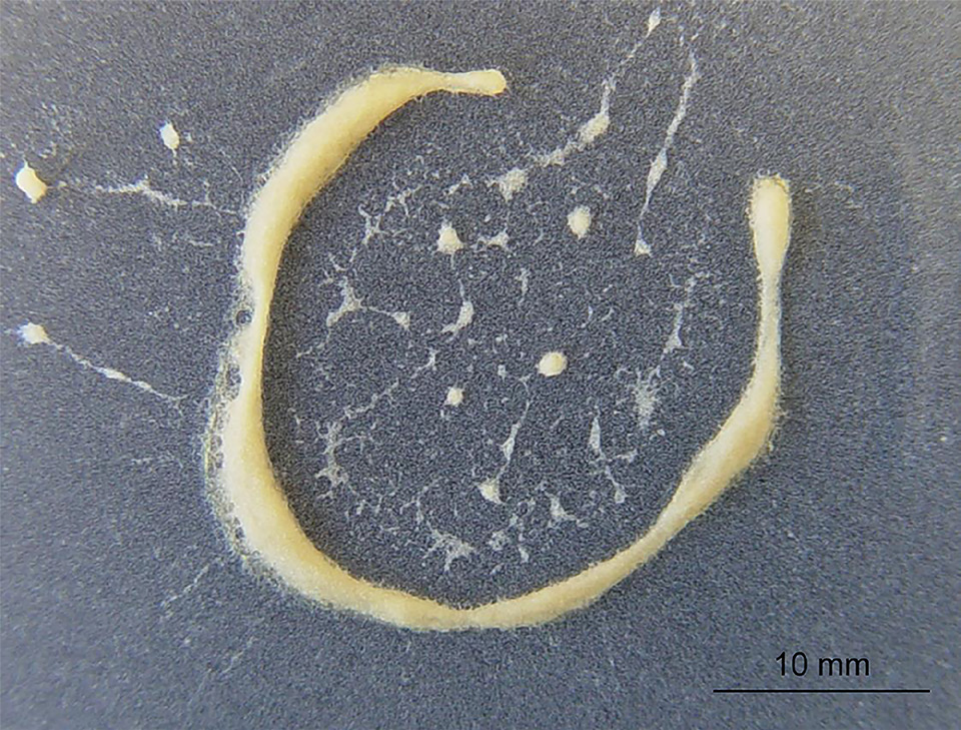
*A. fragariae* aggregated into a compact, dried cluster of “nematode wool” following 24-hr desiccation treatment at 60 ± 2% relative humidity and 23 ± 2°C.

*Aphelenchoides fragariae* were obtained from the Clemson University Nematode Collection where they had been cultured on *Cylindrocladium* sp. in potato dextrose agar (PDA, HiMedia Laboratories, India). They were harvested using a Baermann funnel and resuspended in sterile tap water (Baermann, 1917). A 20 ml suspension of approximately 50,000 nematodes (mixed life stages) was exposed to reduced relative humidity by vacuum filtration onto a 4.7 cm Nuclepore membrane with 5 μm pores (Whatman, Piscataway, NJ). The membrane was transferred to an uncovered petri dish in an airtight glass chamber containing a 72% glycerol solution to maintain a relative humidity of 60 ± 2% (Forney and Brandl, 1992). A MicroRHTemp Data Logger (Madgetech, Warner, NH) was placed in the chamber to collect relative humidity and temperature data, and the chamber was incubated at room temperature (23 ± 2°C) for 24 hr. Nematodes formed dried aggregates or “nematode wool” on the membrane after 24 hr (Fig. 1); these aggregates were collected into a microcentrifuge tube for subsequent RNA isolation. Previous experiments have shown that aggregated *A. fragariae* are capable of rapidly resuming physiological activity upon rehydration (Fu et al., 2012). An equal number of nematodes were maintained for 24 hr in sterile tap water to serve as the fully hydrated control. Given that the number of airtight chambers was a limiting factor, we set up each pair of dehydration and control in three different times, serving as three biological replicates. Nematodes were harvested from three biological replicates of each treatment condition.

### RNA isolation and transcriptome sequencing

Total RNA was extracted from approximately 5,000 nematodes harvested from each biological replicate using the PureLink RNA mini Kit (Life Technologies, Austin, TX) following the manufacturer’s instructions. Total RNA was treated with RNase-Free DNase (Qiagen, Germantown, MD) to remove any contaminating DNA. RNA quality and integrity were verified on an Agilent RNA 6000 Nano LabChip using the Agilent 2100 Bioanalyzer (Agilent Technologies, Santa Clara, CA). Six RNA samples were sent to the Clemson University Genomics Institute (Clemson, SC) for strand-specific, paired-end 125-bp library preparation with the Illumina TruSeq stranded mRNA library kit, followed by sequencing on the Illumina HiSeq 2000 platform (Illumina, San Diego, CA). Raw sequence data were uploaded to the NCBI Sequence Read Archive under accession number SRP148503.

### Transcriptome assembly and annotation

Read quality was assessed with FastQC (https://www.bioinformatics.babraham.ac.uk/projects/fastqc/), followed by adaptor trimming and content-dependent quality trimming with Cutadapt v1.1.2 (quality threshold 20, minimum length 50 bp; Martin, 2011). The average per-read PHRED quality score after trimming and filtering was 36. Trimmed reads from all biological samples were combined for *de novo* transcriptome assembly using default settings of Trinity v2.6.6 (Grabherr et al., 2011; Haas et al., 2013). The set of Trinity contigs with open reading frames of at least 200 base pairs was considered to represent the protein-coding transcriptome. The transcriptome contained 48,541 putative protein-coding genes and 147,621 alternative isoforms of these genes.

The longest isoform per gene was extracted using a utility script bundled with Trinity v2.6.6 (get_longest_isoform_seq_per_trinity_gene.pl). A fasta file of these transcripts is presented as Supplementary File 1, https://doi.org/10.5061/dryad.8pk0p2njc. Functional annotation of the assembled transcripts was performed with Blast2GO 5.0, which executes a blastx search against the NCBI non-redundant database (E  ≤ 1.0^−3^; Pruitt et al., 2006) and assigns GO terms, InterPro IDs, enzyme codes, and KEGG pathways to each transcript (Conesa et al., 2005). Blast2GO annotations of the transcriptome are presented in Supplementary File 2, https://doi.org/10.5061/dryad.8pk0p2njc. Disorder and hydropathy predictions were generated for a subset of unannotated proteins using PONDR with the VSL2 predictor (http://www.pondr.com) and the GRAVY hydropathy calculator (http://www.gravy-calculator.de). Nucleotide sequences of these proteins were also used as blastx queries against the Late Embryogenesis Abundant Protein database (Hunault and Jaspard, 2010).

### Differential gene expression analysis

Trimmed reads from each sample were aligned back to the assembled transcriptome using Bowtie2 (Langmead and Salzberg, 2012), and transcript abundances in each sample were estimated using RSEM (Li and Dewey, 2011). Reads from all splice forms of a given gene were pooled for downstream analysis. Differential gene expression analysis was performed using edgeR, including only those genes that had counts-per-million above 0.5 in least three samples (Robinson et al., 2010; Chen et al., 2016). Genes whose expression differed significantly between desiccated and control samples were identified using the exact test model in edgeR (Robinson et al. 2010; false discovery rate = 0.05). R package pheatmap was used to run hierarchical clustering with “complete” method for a subset of differentially expressed genes (Kolde and Kolde, 2018). Expression data for all genes are presented in Supplementary File 3, https://doi.org/10.5061/dryad.8pk0p2njc.

Gene set enrichment analysis (GSEA v.2.1.0) was performed to identify pre-defined gene sets that showed significant, concordant differences in expression between control and desiccated samples (Mootha et al., 2003; Subramanian et al., 2005). While edgeR identifies individual genes with large, significant fold-changes, GSEA identifies gene sets whose members show concordant, but potentially smaller, changes in expression. A custom GSEA database of 5,988 gene sets, each containing between 5 and 1,500 genes, was created from GO terms and enzyme code annotations of the assembled transcripts. Gene sets whose expression was enriched or depleted in desiccated nematodes were identified using a false discovery rate of 0.05 (Supplementary File 4, https://doi.org/10.5061/dryad.8pk0p2njc).

## Results and discussion

### Transcriptome sequencing and de novo assembly

**Figure S1:**
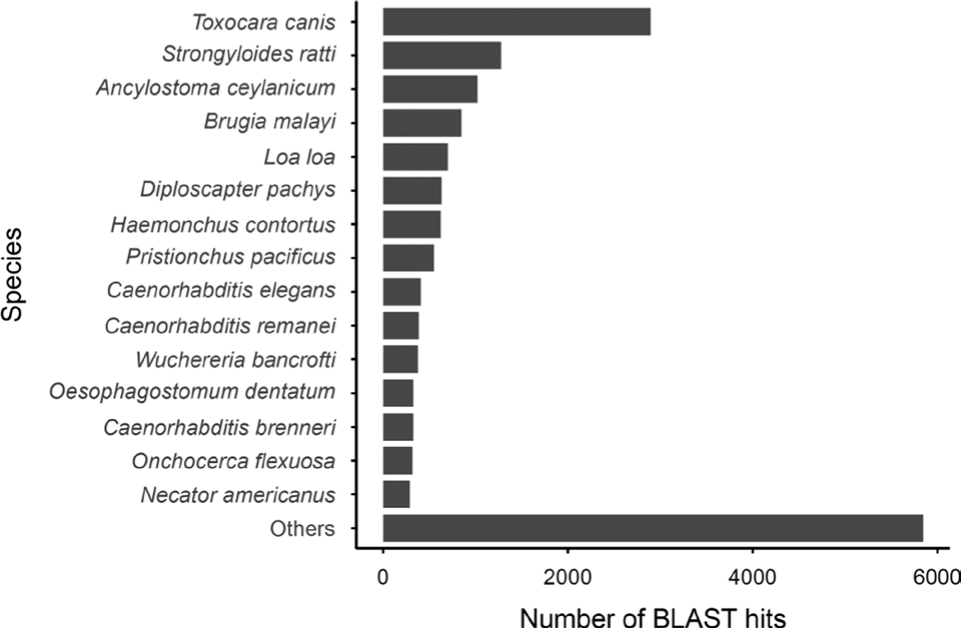
Top blastx hit species for 48,541 putative protein-coding genes from *Aphelenchoides fragariae*.

Illumina sequencing of RNA samples from desiccated and control nematodes generated 325 million reads with a mean length of 125 bp and an average GC content of 42%. After filtering and trimming, reads from all samples were combined for *de novo* assembly with the Trinity pipeline. The final protein-coding transcriptome contained 48,541 putative protein-coding genes with 147,621 alternate splice forms and an N50 of 1293 bp (Table S1). In all, 35% of the assembled genes had at least one hit against the NCBI nr database, and 23% were annotated with at least one GO term in Blast2GO (Table S1). The most common top hit species were *Toxocara canis*, *Strongyloides ratti*, and *Ancylostoma ceylanicum*, all of which are fully sequenced animal parasitic nematodes (Fig. S1 ).

**Table S1.** Summary statistics for *Aphelenchoides fragariae* transcriptome assembly.

Basic sequence statistics	Number
Total raw reads	324,895,970
Mean read length (bp)	125
Raw read GC content	42%
Mean read PHRED score after filtering and trimming	36
Number of genes	48,541
Number of isoforms	147,621
Assembly N50 (of all isoforms)	1293 bp
Ex90N50	1470 bp
Mean length of all isoforms	882 bp
Top BLASTx-hit species	*Toxocara canis*
Percent of gene with at least one BLASTx hit (E ≤ 1.0-3)	35%
Percent of gene with at least one GO annotation	23%

### Upregulation of detoxification-related gene and gene sets

**Figure 2:**
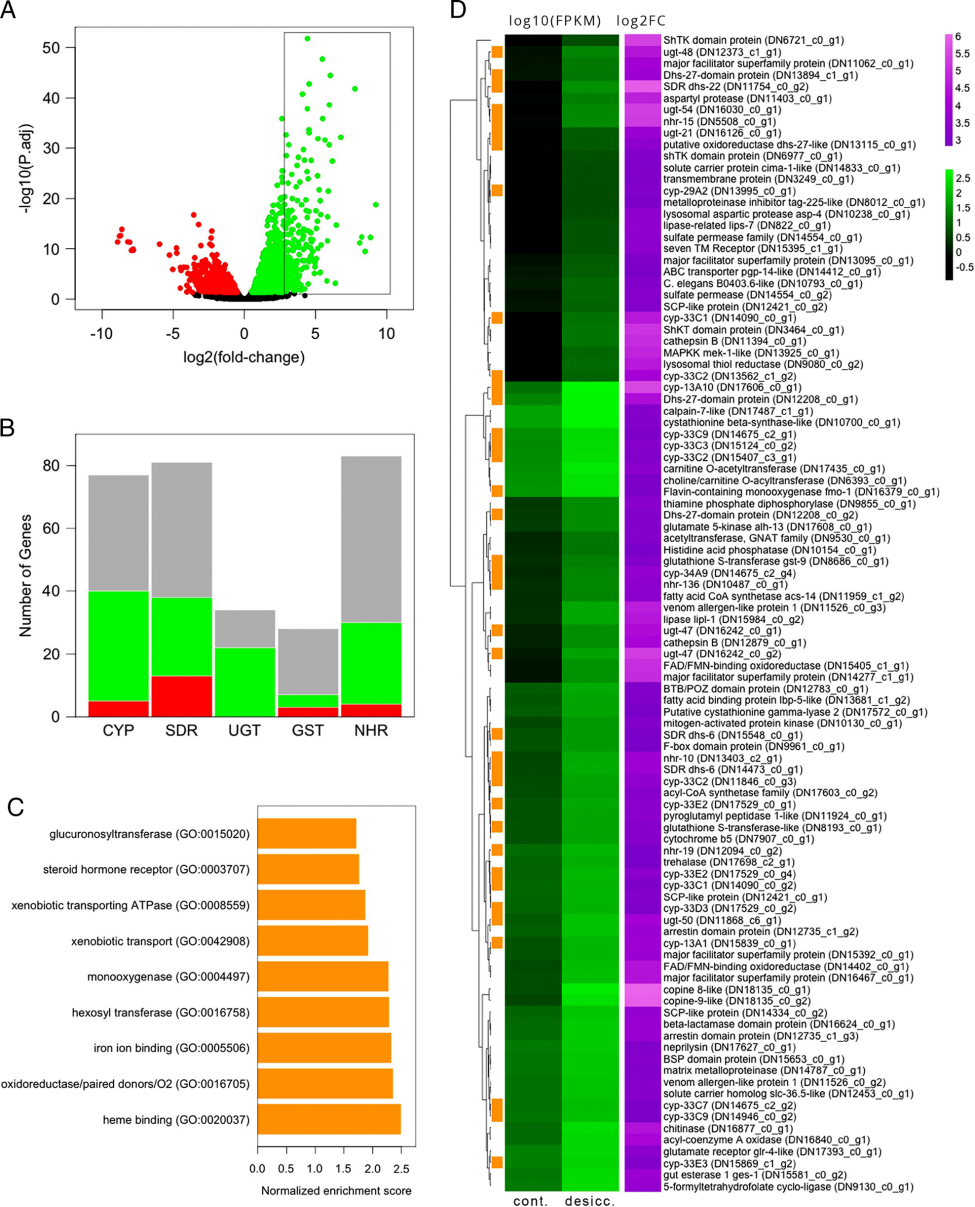
A: Volcano plot of differentially expressed genes between control and desiccated *A. fragariae*. Black: genes that were not differentially expressed; green: upregulated genes in desiccation; red: downregulated genes in desiccation (FDR = 0.05). Box encloses 100 genes with the largest positive fold changes; B: Number of *A. fragariae* genes from five families with documented roles in detoxification: cytochrome P450s (CYP), short chain dehydrogenase/reductases (SDR), UDP-glucuronosyltransferases (UGT), glutathione-S-transferases (GST), and nuclear hormone receptors (NHR). Green: genes upregulated in desiccation; red: genes downregulated in desiccation; grey: genes that were not differentially expressed (FDR = 0.05); C: Nine gene sets, defined based on shared gene ontology terms, whose expression was significantly enriched under desiccation (FDR = 0.05). Orange bar: normalized enrichment score for each set (Subramanian et al., 2005); D: Heat map of log_10_(FPKM) values in green and log_2_(fold-change) values in purple for 100 genes with the largest positive fold-changes in response to desiccation. Genes marked with orange bars have putative roles in detoxification.

In total, 2,083 and 953 genes were significantly up- and downregulated, respectively, in desiccated nematodes (Fig. 2A). Of the 100 annotated genes with the largest positive fold-changes (box, Fig. 2A), more than one third encoded putative detoxification-related proteins (Fig. 2D). These included numerous Phase I and II detoxification enzymes: CYPs, SDRs, UGTs, and GSTs. Also among the top hundred upregulated genes were a *pgp-14*-like multi-drug resistance protein (MRP/PGP), an FAD-dependent monooxygenase, and multiple nuclear hormone receptors (NHRs), all of which have been implicated in the cellular detoxification program (Fig. 2; Lindblom and Dodd, 2006; Hodge and Tracy, 2010; Adhikari and Adams, 2011; Hoffmann and Partridge, 2015; Harder, 2016).

Across the transcriptome as a whole, large percentages of CYPs (45%), SDRs (32%), UGTs (55%), NHRs (30%), and MRP/PGPs (32%) were upregulated in response to desiccation (Fig. 2B). Only the GSTs did not respond strongly as a group: 28 GST genes were assembled: 5 (18%) were significantly upregulated and three were downregulated.

Results of gene set enrichment analysis were consistent with wholesale induction of detoxification-related genes. Of the top 15 gene sets enriched during desiccation, 5 were dominated by detoxification-related genes. The majority of leading-edge genes in each set (i.e. those that contributed to the enrichment signal) were again CYPs, UGTs, NHRs, and MRP/PGPs. Gene sets associated with other detoxification-related GO terms were also significantly enriched (Fig. 2C): xenobiotic transport (GO:0042908), xenobiotic transporting ATPase (GO:0008559), steroid hormone receptor (GO:0003707), and glucuronosyltransferase (GO:0015020). The leading-edge genes of the former two sets were made up entirely of MRP/PGP genes. The leading-edge genes for GO:0003707 were entirely NHRs, while those of GO:0015020 were primarily UGTs.

CYPs and SDRs are canonical enzymes of Phase I detoxification that add reactive functional groups to a wide variety of endogenous and exogenous compounds. Both are large gene families: 86 CYPs and 68 SDRs have been documented in *C. elegans*, and 80 CYPs and 84 SDRs were expressed here in *A. fragariae* (Lindblom and Dodd, 2006). CYPs primarily hydroxylate lipophilic substrates, including endo- and xenobiotics, steroids, and fatty acids. SDRs also activate lipophilic substrates by catalyzing the reduction of carbonyl groups in aldehydes and ketones. A number of strongly upregulated SDRs in *A. fragariae* were similar to the uncharacterized *C. elegans*’ short chain dehydrogenase *dhs-27*, an ortholog of human HSDL1 (hydroxysteroid dehydrogenase like 1). The reactions catalyzed by CYPs and SDRs are energetically expensive, consuming NADH/NADPH during substrate functionalization (Gems and McElwee, 2005).

Functionalized substrates are further modified by the Phase II reactions catalyzed by UGTs and GSTs. These reactions typically involve the addition of side groups that increase the substrate’s solubility in preparation for excretion. Like CYPs, UGTs act on numerous small lipophilic compounds: xenobiotics, endogenous waste metabolites, steroids, and fatty acids. GSTs participate in the modification and detoxification of substrates by multiple mechanisms, including the addition glutathione to an electrophilic substrate and the direct binding of toxic substrates. These are also expensive reactions: each UGT glucuronidation reaction requires one molecule of glucose, while each GST transferase reaction uses one molecule of reduced glutathione (Gems and McElwee, 2005; Lindblom and Dodd, 2006). Following enzymatic modification by Phase I and II enzymes, toxins and waste metabolites are excreted from the cell by membrane transporters, mainly members of the ATP-binding cassette (ABC) family of efflux pumps (Lindblom and Dodd 2006). Among this large protein family, MRP and PGP transporters have been most extensively characterized for their role in detoxification (Choi, 2005; Hoffmann and Partridge, 2015; Harder, 2016).

NHRs are a very large class of transcription factors (over 280 in *C. elegans*). In most cases, NHRs bind lipophilic hormones, e.g. steroids, retinoids, and fatty acids, and also regulate gene expression through the integration of endogenous and exogenous signals. Specific NHRs, such as NHR-8 and NHR-48 in *C. elegans* and DHR96 in *Drosophila*, regulate xenobiotic detoxification network in response to environmental conditions (Hoffmann and Partridge, 2015). A gene (DN12692_c0_g8) in our data set that is homologous to *nhr-48* was upregulated despite its low expression. Additionally, DAF-12 (*C. elegans*), NHR-8, and DHR96 are involved in metabolism of steroid hormone, cholesterol, and triacylgylceride, which are related to healthy aging (King-Jones et al., 2006; Bujold et al., 2010; Horner et al., 2009; Antebi, 2013; Magner et al., 2013; Wang et al., 2015). Desiccated nematodes undergo extended life span, and it is critical to maintain health during such process. Our data have shown that genes that are key to xenobiotic detoxification network were activated in desiccated nematodes, suggesting the connection between desiccation and healthy aging via detoxification.

The broad induction of detoxification-related genes was the single most striking pattern to emerge from the desiccation-related transcriptome. This result is intriguing, as it parallels the broad induction of detoxification-related gene expression that has been reported in *C. elegans*’ stress-tolerant, growth-arrested dauer larvae and in the long-lived *C. elegans daf-2* mutant (Gems and McElwee, 2005; Erkut et al., 2013). Both dauers and long-lived mutants upregulate CYPs, SDRs, UGTs and GSTs (McElwee et al., 2004). Evidence suggests that induction of the detoxification program may occur broadly in response to dehydration: several previous studies of anhydrobiotic organisms have noted upregulation of detoxification-related genes. Desiccation-related ESTs from the Antarctic nematode, *Plectus murrayi*, included a GST, an NHR, an aldehyde dehydrogenase, and several ABC transporters. Among the most highly represented KEGG pathways in the EST library was “xenobiotic and bio-degradation” (Adhikari et al., 2009; Adhikari and Adams, 2011). A CYP was one of 15 dehydration-responsive genes identified by suppressive subtractive hybridization in the Antarctic midge, *Belgica antarctica* (Lopez-Martinez et al., 2009), and multiple CYPs and GSTs were induced by dehydration in the dog tick, *Dermacentor variabilis*.

This raises the question of what, exactly, is being detoxified in stress tolerant and/or long-lived organisms. Anhydrobitic *A. fragariae* and *C. elegans* dauers do not feed, it is therefore unlikely that detoxification reactions are required to process exogenous xenobiotic toxins. It has been suggested that these detoxification reactions are used to eliminate accumulated endogenous lipophilic wastes, the products of stochastic errors in metabolism or stress-related damage (Gems and McElwee, 2005). Alternately, detoxification enzymes are also involved in hormone and fatty acid metabolism, and these functions may be the principle means by which they influence stress tolerance, development and longevity (Zimniak, 2008; Hodges and Minich, 2015). These potential roles—clearing of endogenous lipophilic waste, hormonal regulation of development, and control of fatty acid metabolism—are not mutually exclusive. All may play a part in survival and longevity. Detoxification reactions are energetically expensive, and the fact that detoxification-related gene expression is prioritized under non-feeding, stressful conditions suggests detoxification, while costly, is vital to survival during and/or after release from anhydrobiosis.

### Heat shock proteins, chaperones, and the unfolded protein response

**Figure 3:**
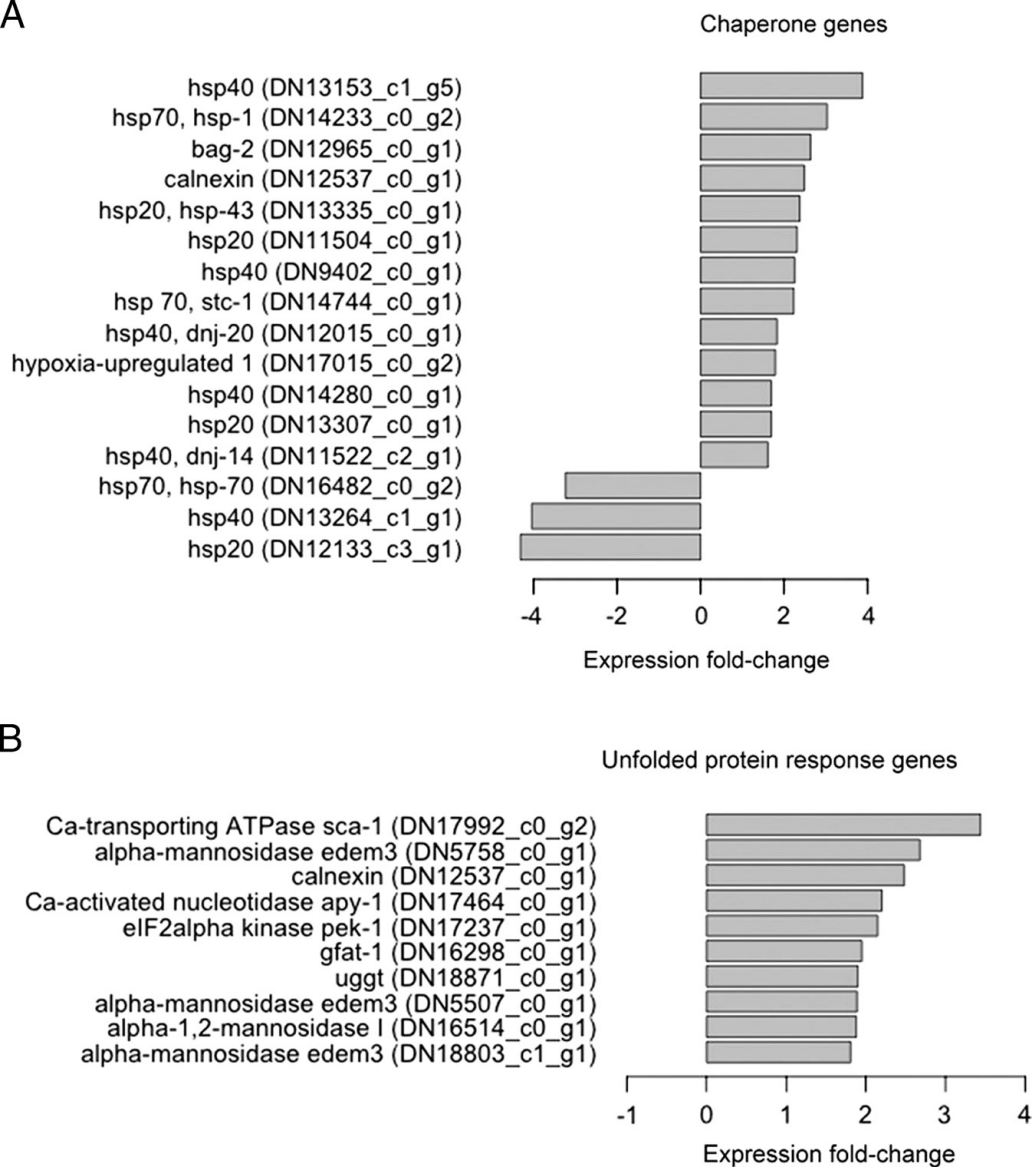
Differentially expressed genes of *A. fragariae* that function as chaperones in A and respond to unfolded proteins in B.

Desiccation can cause protein misfolding, damage, and aggregation (Tapia et al., 2015). Indeed, such damaged proteins may be important substrates for the detoxication enzymes highlighted above, such as CYP, UGT, SDR, and NHR. Multiple genes encoding molecular chaperones and components of the unfolded protein response were induced by desiccation in *A. fragariae* (Fig. 3). These molecular chaperones support proper protein folding and reduce protein aggregation by both ATP-dependent and ATP-independent mechanisms (Hartl et al., 2011; Basha et al., 2012).

Of the 98 heat shock protein genes assembled from *A. fragariae*, 11 were upregulated and three were downregulated (Fig. 3A). Upregulated genes encoded chaperones from the hsp20, hsp40, and hsp70 families. It should be noted that many *hsp* genes were expressed at high levels under both desiccated and control conditions. For example, four hsp90 genes and three hsp70 genes were expressed at levels greater than 1000 FPKM under both conditions (Supplementary File 3, https://doi.org/10.5061/dryad.8pk0p2njc). Also significantly upregulated by desiccation were the molecular chaperones *bag-2* and calnexin (Fig. 3A). The latter is an endoplasmic reticulum (ER) chaperone that functions in the unfolded protein response (Takayama et al., 1999; Lee et al., 2005).

Desiccated *A. fragariae* also showed upregulation of additional unfolded protein response genes and enriched expression of related gene sets (Fig. 3B). Upregulated genes included UGGT (UDP-glucose glycoprotein glucosyltransferase), which adds glucose residues to misfolded glycoproteins; alpha-1,2-mannosidase, which removes mannose residues from misfolded proteins; and alpha-mannosidase EDEM3, which trims mannose residues and directs the trimmed proteins to the ER-associated degradation pathway (Sousa and Parodi, 1995; Hosokawa et al., 2001; Hirao et al., 2006). Finally, *pek-1*, whose encoded protein globally suppresses translation during ER lumen stress, was also induced by desiccation (Richardson et al., 2011).

### Intrinsically disordered proteins

Another means by which dehydrating organisms may prevent catastrophic damage to proteins and membranes is through the production of strongly hydrophilic, IDPs. These proteins have been documented in anhydrobiotic species from multiple kingdoms of life. Often classified as late embryogenesis abundant (LEA) proteins, IDPs are thought to stabilize proteins, membranes, and organelles during desiccation (Hand et al., 2011). Recently, novel desiccation-induced IDPs with no homology to other known proteins were identified in tardigrades and shown to mediate desiccation tolerance (Boothby et al., 2017).

A subset of strongly upregulated *A. fragariae* transcripts appeared to code for IDPs. The putative *A. fragariae* IDP transcripts received no annotations in the Blast2GO pipeline despite possessing open reading frames longer than 200 bp and substantial read support. Their fold-changes and absolute FPKM values were high, and some received significant blastx hits to the LEA Protein database. Most contained tandem repeats of short amino acid motifs, a common feature of IDPs (Jorda et al., 2010). All were enriched in disorder-promoting amino acid residues such as glycine, serine, lysine, and glutamine (Uversky, 2019). In total, 14 such transcripts are presented in Table 1, with information on their length, predicted disorder, hydropathy, and blastx hits to known LEA proteins.

**Table 1.** Characteristics of 14 putative intrinsically disordered proteins whose expression was significantly upregulated under desiccation in *Aphelenchoides fragariae*.

Gene ID	Predicted length (aa)	Mean FPKM desiccated	Mean FPKM control	Fold-change	Adjusted *P-*value	% disordered residues	GRAVY hydropathy value	Blastx hits to LEA database
DN15064_c0_g1	175	398.8	0.4	1020.9	3.15E^−64^	88.1	−1.836	−
DN14203_c3_g3	119	45.4	0.5	88.7	1.92E^−15^	74.8	−0.977	−
DN10042_c0_g2	128	366.8	5.0	72.9	3.61E^−28^	82.8	−0.108	*Sophora davidii* dehydrin DHN, E = 2e^−13^
DN10455_c1_g1	191	928.2	14.0	64.1	9.30E^−99^	90.6	−0.503	*Sophora davidii* dehydrin DHN, E = 8e^−09^
DN9957_c2_g1	335	7.9	0.2	41.1	1.66E^−17^	94.6	−1.104	*Sorghum bicolor* dehydrin-like SORBIDRAFT_10g003700, E = 2e^−14^
DN10923_c0_g1	171	17.0	0.7	25.5	1.23E^−12^	81.9	−1.607	*Trifolium repens* dehydrin b, E = 0.002
DN9863_c0_g1	250	21.7	0.9	23.4	1.70E^−43^	85.2	−0.951	*Arabidopsis thaliana* dehydrin rab18, E = 2e^−10^
DN9710_c0_g1	167	28.1	1.3	20.6	4.50E^−07^	93.5	−1.185	−
DN12711_c2_g2	131	92.8	6.4	13.8	4.35E^−12^	77.1	−0.204	*Sophora davidii* dehydrin DHN, E = 7e^−07^
DN11488_c0_g1	192	16.40	1.30	11.50	2.52E^−03^	86.5	−1.167	−
DN12459_c0_g4	172	173.6	14.4	11.5	7.31E^−17^	98.3	−0.793	*Eucalyptus grandis* dehydrin 1, E = 5e^−07^
DN15965_c0_g1	140	294.5	25.7	10.7	2.28E^−10^	72.9	−0.450	*Hordeum vulgare* dehydrin dhn4, E = 8e^−12^
DN12325_c1_g1	96	15.5	1.9	8.1	1.38E^−17^	75.0	−0.672	−
DN9469_c2_g1	133	46.8	9.7	4.7	1.44E^−14^	100.0	−1.520	*Phaseolis vulgaris* dehydrin PHAVU_009G004400g, E = 3e^−05^

It appeared that many aspects of the desiccation response and network of genes are coordinated by a transcriptional factor *daf-16*, which was upregulated four-fold in desiccated *A. fragariae.* In *C. elegans*, *daf-16* encodes a forkhead box O transcription factor that functions as the central regulator of insulin/IGF-1 signaling (IIS) pathway (Tullet, 2015; Sun et al., 2017). In addition, *daf-16* integrates other signals pathways that module aging and longevity, such as target of rapamycin and AMP-activated protein kinase (Sun et al., 2017). *Daf-16* activity is normally repressed by the food-sensing insulin/IGF signaling pathway (McElwee et al., 2003). In *daf-2* mutant *C. elegans*, of which worm’s longevity is doubled, de-phosphorylation of *daf-16* leads to localization and accumulation of *daf-16* in the nucleus. In turn, *daf-16* could regulate a collection of genes involved in stress resistance, fat metabolism, defense against pathogens, regulation of dauer formation, and pathways that influence life span (Murphy 2006; Tullet 2015). Many genes or homolog of the genes that are regulatory targets of *daf-16*, e.g. GSTs, CYP, SDR, UGT (McElwee et al., 2004), and small heat shock proteins (Murphy, 2006), exhibited upregulation pattern in our study, suggesting the central role of IIS and possible integration of other signaling pathways are associated to desiccation in foliar nematodes. Under anhydrobiotic state where many metabolic activities are suspended, it is no surprise that desiccation shares similar pathways and gene regulatory networks that are related to healthy aging and extended life span.

It is important to note that nematodes in our experiment were sampled at only one time point, 24 hr after relatively rapid and severe dehydration. While the desiccated worms were fully viable and capable of recovery within 30 min of rehydration (Fu et al., 2012), their transcriptomes likely reflected both the remnants of transcriptional programs induced early in desiccation and those active in later stages of desiccation. In the future, a more detailed time course of the transcriptional changes that accompany desiccation and rehydration could clarify the order in which specific gene expression changes and signaling events occur.

Anhydrobiosis is more than just a biological curiosity: the conservation of its basic biochemical mechanisms across multiple kingdoms of life – from bacteria to plants to arthropods – suggests that the ability to tolerate significant dehydration is both ancient and conserved. Anhydrobiotic physiology exhibits numerous connections with the basic biology of longevity and aging. A better understanding of anhydrobiosis therefore has genuine implications for human health and lifespan, some of which are already being explored in clinical settings (Crowe, 2015). From an agricultural standpoint, induction of anhydrobiotic stasis offers a means of stabilizing and delivering living amendments such as entomopathogenic nematodes and fungi as biocontrol, while interruption or elimination of anhydrobiosis could disrupt the life cycle of damaging pests such as cyst nematodes and *A. fragariae*. Manipulation of organisms’ anhydrobiotic physiology will require a more detailed understanding of its underlying mechanisms and the extent to which they are conserved across species.
